# Supplementary material for: Effectiveness of the Combined Use of a Brain–Machine Interface System and Virtual Reality as a Therapeutic Approach in Patients with Spinal Cord Injury: A Systematic Review
Source: Healthcare (Basel). 2023 Dec 17;11(24):3189. doi: 10.3390/healthcare11243189 (PMC10742447; doi:10.3390/healthcare11243189)
Supplement: Supplementary file 1 [file healthcare-11-03189-s001.zip › healthcare-2711934-supplementary/Figure S2. Pubmed Database Search..pdf]

Figure S2. Pubmed Database Search

History and Search Details

Download Delete

Search	Actions	Details	Query	Results	Time
#10	•••	›	Search: **#8 OR #9** Filters: **Clinical Trial, Humans, English**	6	04:12:20
#9	•••	›	Search: **#6 AND #7** Filters: **Clinical Trial, Humans, English**	0	04:11:41
#8	•••	›	Search: **#1 AND #5** Filters: **Clinical Trial, Humans, English**	6	04:11:09
#7	•••	›	Search: **"Brain computer interfaces"[MeSH Terms]** Filters: **Clinical Trial, Humans, English**	123	04:08:43
#6	•••	›	Search: **"video games"[MeSH Terms]** Filters: **Clinical Trial, Humans, English**	992	04:07:41
#5	•••	›	Search: **"virtual reality"[MeSH Terms]** Filters: **Clinical Trial, Humans, English**	617	04:06:54
#4	•••	›	Search: **"virtual reality"[MeSH Terms]** Filters: **Humans, English**	4,567	04:06:48
#3	•••	›	Search: **"virtual reality"[MeSH Terms]** Filters: **Humans**	4,687	04:06:44
#2	•••	›	Search: **"virtual reality"[MeSH Terms]**	4,986	04:06:37
#1	•••	›	Search: **"spinal cord injuries"[MeSH Terms]** Filters: **Clinical Trial, Humans, English**	2,165	04:04:39

Showing 1 to 10 of 10 entries

1: Richardson EJ, McKinley EC, Rahman AKMF, Klebine P, Redden DT, Richards JS. Effects of virtual walking on spinal cord injury-related neuropathic pain: A randomized, controlled trial. Rehabil Psychol. 2019 Feb;64(1):13-24. doi: 10.1037/rep0000246. Epub 2018 Nov 8. PMID: 30407030.

2: Lakhani A, Martin K, Gray L, Mallison J, Grimbeek P, Hollins I, Mackareth C. What Is the Impact of Engaging With Natural Environments Delivered Via Virtual Reality on the Psycho-emotional Health of People With Spinal Cord Injury Receiving Rehabilitation in Hospital? Findings From a Pilot Randomized

Controlled Trial. Arch Phys Med Rehabil. 2020 Sep;101(9):1532-1540. doi: 10.1016/j.apmr.2020.05.013. Epub 2020 Jun 2. PMID: 32502564.

3: Austin PD, Craig A, Middleton JW, Tran Y, Costa DSJ, Wrigley PJ, Siddall PJ. The short-term effects of head-mounted virtual-reality on neuropathic pain intensity in people with spinal cord injury pain: a randomised cross-over pilot study. Spinal Cord. 2021 Jul;59(7):738-746. doi: 10.1038/s41393-020-00569-2. Epub 2020 Oct 19. PMID: 33077900.

4: Tran Y, Austin P, Lo C, Craig A, Middleton JW, Wrigley PJ, Siddall P. An Exploratory EEG Analysis on the Effects of Virtual Reality in People with Neuropathic Pain Following Spinal Cord Injury. Sensors (Basel). 2022 Mar 29;22(7):2629. doi: 10.3390/s22072629. PMID: 35408245; PMCID: PMC9002545.

5: Dimbwadyo-Terrer I, Trincado-Alonso F, de Los Reyes-Guzmán A, Aznar MA, Alcubilla C, Pérez-Nombela S, Del Ama-Espinosa A, Polonio-López B, Gil-Agudo Á. Upper limb rehabilitation after spinal cord injury: a treatment based on a data glove and an immersive virtual reality environment. Disabil Rehabil Assist Technol. 2016 Aug;11(6):462-7. doi: 10.3109/17483107.2015.1027293. Epub 2015 Jul 16. PMID: 26181226.

6: Duffell LD, Paddison S, Alahmary AF, Donaldson N, Burridge J. The effects of FES cycling combined with virtual reality racing biofeedback on voluntary function after incomplete SCI: a pilot study. J Neuroeng Rehabil. 2019 Nov 27;16(1):149. doi: 10.1186/s12984-019-0619-4. PMID: 31771600; PMCID: PMC6880599.
