# Supplementary material for: Effectiveness of the Combined Use of a Brain–Machine Interface System and Virtual Reality as a Therapeutic Approach in Patients with Spinal Cord Injury: A Systematic Review
Source: Healthcare (Basel). 2023 Dec 17;11(24):3189. doi: 10.3390/healthcare11243189 (PMC10742447; doi:10.3390/healthcare11243189)
Supplement: Supplementary file 1 [file healthcare-11-03189-s001.zip › healthcare-2711934-supplementary/Table S1. PEDro scores obtained of the randomized controlled trials included in the systematic review..pdf]

Table S1. PEDro scores obtained of the randomized controlled trials included in the systematic review.

Study	1	2	3	4	5	6	7	8	9	10	11	Total
**Nicolelis et al., 2022 [40]**	-	YES	NO	YES	NO	NO	YES	YES	YES	YES	YES	**7**

Range: 0–10. Item 1 is not used in the method score.

Note. "YES" indicates that a study meets that particular criterion. "NO" means that this study does not meet the criteria or that it does not provide enough information to be sure. 1. eligibility criteria were specified; 2. subjects were randomly allocated to groups (in a crossover study, subjects were randomly allocated an order in which treatments were received); 3. allocation was concealed; 4. the groups were similar at baseline regarding the most important prognostic indicators; 5. there was blinding of all subjects; 6. there was blinding of all therapists who administered the therapy; 7. there was blinding of all assessors who measured at least one key outcome; 8. measures of at least one key outcome were obtained from more than 85% of the subjects initially allocated to groups: 9. all subjects for whom outcome measures were available received the treatment or control condition as allocated or, where this was not the case, data for at least one key outcome was analyzed by "intention to treat"; 10. the results of between-group statistical comparisons are reported for at least one key outcome; 11. the study provides both point measures and measures of variability for at least one key outcome.
